# Assessing medication packaging and labelling appropriateness in Sri Lanka

**DOI:** 10.1186/s40545-016-0091-5

**Published:** 2016-11-25

**Authors:** N. Athuraliya, E. J. Walkom, S. Dharmaratne, J. Robertson

**Affiliations:** 1 Department of Medicine, The Maitland Hospital Clinical School, 550-560 High Street, Maitland, 2320 NSW Australia; 2 Department of Clinical Pharmacology, School of Medicine and Public Health, The University of Newcastle, Newcastle, Australia; 3 Medical Education Unit, Faculty of Medicine, University of Peradeniya, Peradeniya, Sri Lanka

**Keywords:** Medicines, Packaging, Labelling, Dispensing, Pharmacy practice, Low-middle income countries

## Abstract

**Background:**

There is substantial evidence of poor dispensing practices with inadequate packaging and labelling of medicines, and limited advice on their usage in low and middle-income countries (LMICs). We examined the labelling and packaging of medicines identified during a survey of 1322 households in six regions of Sri Lanka between 2010 and 2013 conducted using the World Health Organization (WHO) methodology for household surveys. We compared medicines obtained from public and private sources and asked interviewees if they understood how to take the medicines.

**Methods:**

Packaging was considered adequate when the primary package was an envelope or closable container holding only one medicine. Adequate labels were legible and included medicine name, dose and expiration date. Interviewers assessed whether respondents knew how to take the medicines.

**Results:**

Of 1322 households, 1253 households (94.8%) had at least one medicine; 84% were classified as western medicines and 16% traditional medicines. Of 5756 western medicines identified, 82.1% were adequately packaged, 43.3% adequately labelled and 41.4% both adequately packaged and labelled. Participants stated that they understood the label and knew how to take 96% of the medicines. Private medicine sources had more adequately packaged medicines than public sources (87.7% vs 73.5%; OR 2.58, 95% CI 2.23, 2.99) and more adequately labelled medicines (52.2% vs 27.4%; OR 2.90, 95% CI 2.57, 3.26).

**Conclusions:**

Inadequate packaging and labelling of medicines remain a concern in Sri Lanka. Commitment to Good Pharmacy Practices, investments in staff education and training and adequate dispensing resources (containers and labels), particularly in the public sector, are needed to address sub-optimal dispensing practices. Ageing populations with more chronic diseases requiring polypharmacy and complex medicine regimens increase the need for appropriately packaged and labelled medicines.

**Electronic supplementary material:**

The online version of this article (doi:10.1186/s40545-016-0091-5) contains supplementary material, which is available to authorized users.

## Background

Medicines are central to health care and the most commonly used therapeutic intervention to manage acute and chronic conditions. The rational or responsible use of medicines depends on an appropriate clinical diagnosis and selection of the appropriate medicines in the correct dose and duration at the lowest cost to patients [1]. Good dispensing practices ensure that the medicine is delivered to the patient with clear instructions in a package that maintains the potency of the medicine up to the time of use [2]. Desirable and acceptable forms of packaging for different pharmaceutical dosage forms have been defined, however the high costs and poor availability of suitable containers can compromise dispensing practices in some settings [2].

Medicine containers should provide a surface for attaching or writing a label with identifying details and instructions for use. In addition, dispensers have a responsibility to ensure that patients understand how to take their medicines [2]. However, there is substantial evidence of poor dispensing practices, inadequate packaging, labelling, instruction and advice on usage and, storage of medicines in low and middle-income countries (LMICs) [3].

Country situational analyses conducted in South East Asia in Bhutan, India, Indonesia, Myanmar, Nepal, and Timor-Leste have documented the frequent use of small plastic bags as packaging, sometimes writing the number of tablets per day and dosage frequency on a separate, unattached slip of paper, or no labelling of dispensed medicines at all [4]. Studies conducted in Egypt, Northern Nigeria, Pakistan and Ethiopia have reported between 0 and 13.7% of dispensed medicines with adequate labels [5–8]. Yet in these same studies 55 to 94% of patients were reported to have adequate knowledge of the correct dose of the medicine.

In many LMICs the private sector is an important source of medicines, and may be the first point of contact with the health care system and preferred channel for purchasing medicines [3]. However, questions have been raised about the quality of services provided. There is evidence of a lack of pharmacists or other trained personnel in these facilities, provision of advice that is not in accord with treatment guidelines, inappropriate supply of medicines and insufficient counselling [3, 9, 10].

The aim of this study was to examine the situation in Sri Lanka with regards to medicines labelling and packaging and to determine if there were differences in dispensing practices between public and private sources of medicines. In addition, we assessed interviewees’ knowledge of how to take the medicines identified.

## Methods

We conducted a study of access to medicines for acute and chronic illness in 2010/2011 (Kandy district) and 2012/13 (Colombo, Ampara, Monaragala, Polonnaruwa and Rathnapura districts), using the World Health Organization (WHO) methodology for household surveys [11]. Six administrative divisions (districts) were chosen from the 25 districts of Sri Lanka. An initial feasibility study was conducted in Kandy district. Colombo (capital) district was chosen as per the WHO guidelines. The remaining districts were divided into clusters based on the poverty level criteria, childhood anaemia, population density, and percentage of rural and urban populations within the district and selected to ensure inclusion of districts of different socio-economic status.

Thirty public health care facilities distributed across the six districts were randomly selected. The study population consisted of clusters of households at a given distance (<5, 5–10 and >10 km) from the reference health facilities. Thirty households in six clusters around each reference health care facility are recommended to be interviewed, i.e. a national sample of 900 households.

We used six pairs of interviewers who were Sociology, Biology or Pharmacy graduates and trained to collect the information. Training was guided by the WHO training tools (WHO Manual for household surveys; PowerPoint slide presentation). Supervision of interviewers and review of completed questionnaires were undertaken by two of the researchers (SD, NA).

As part of the data collection for household surveys, participants were asked by the interviewers to show all the medications present in their home at the time of the interview. Details were recorded for up to 20 medicines per household. The medicine was identified where possible and respondents were asked where they obtained the medicine: family/friend; public hospital; NGO/mission hospital; public health centre or dispensary; private health care provider; traditional healer; private pharmacy; drug seller and ‘Other’. Medicines were classified as western or traditional. We report here on packaging and labelling of medicines obtained in the public and private sector, i.e. public hospital; public health centre or dispensary; private health care provider; private pharmacy. The labelling of traditional medicines is more complex and less subject to regulation and standardization, therefore a detailed analysis of the packaging and labelling of traditional medicines was beyond the scope of this study.

WHO household study definitions of adequate packaging and labelling were applied. The primary packaging for the medication was assessed and considered adequate if the package was an envelope or a closable container, and contained only one medicine. The interviewer recorded whether the label on the medicines was legible (included medicine name, dose and expiration date), and also whether the interviewee could understand the label and how to take the medicine.

Descriptive statistics (%) are used to summarise results. Differences between groups are presented as Odds Ratios (OR) with 95% confidence intervals (CI) and were calculated using StatsDirect Version 3 (2016).

## Results

There were 1322 households from six regions of Sri Lanka included in the study. Most households had at least one medication to show the interviewer (94.8%, average 5 medicines per household, 4 western and 1 traditional). A total of 6856 medicines were identified; 84% (5756) were classified as western medicines and 16% (1085) traditional medicines (Table 1). Data used to conduct this analysis are available in Additional File 1.

**Table 1:** Household characteristics and medicines by surveyed district

	Ampara	Colombo	Kandy	Monaragala	Polonnaruwa	Rathnapura	Total
Households surveyed (*N*)	216	213	210	216	255	212	1322
Persons per household (mean, range)	4.3 (1–9)	4.3 (1–8)	4.4 (1–9)	4.1 (1–9)	4.1 (1–8)	4.2 (1–9)	4.2 (1–9)
Western medicines (*N*)	765	1074	980	989	847	1101	5756
Western medicines per household (mean, range)	3.5 (0–14)	5.0 (0–15)	4.7 (0–15)	4.6 (0–15)	3.3 (0–15)	5.2 (0–14)	4.4 (0–15)
Traditional medicines per household (mean, range)	0.8 (0–5)	0.9 (0–5)	0.5 (0–5)	1.3 (0–7)	0.7 (0–9)	0.8 (0–4)	0.8 (0–9)

Of the 5756 western medicines, the majority (*n* = 4725, 82.1%) were deemed adequately packaged, while less than half of the medicines assessed (43.3%) were adequately labelled. Only 2385/5756 (41.4%) medicines had both adequate packaging and labelling. Participants reported understanding the instructions for using almost all medicines (95.6%).

**Fig. 1:**
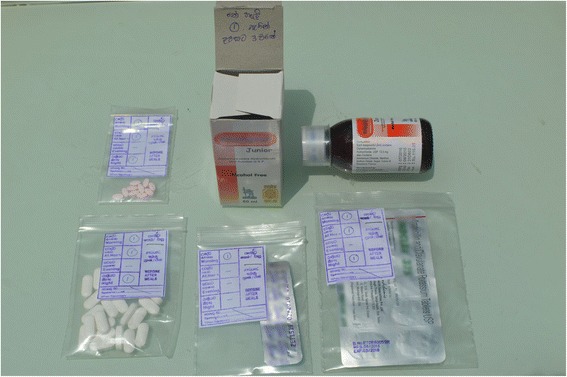
Example of medicines sourced from private facilities

**Fig. 2:**
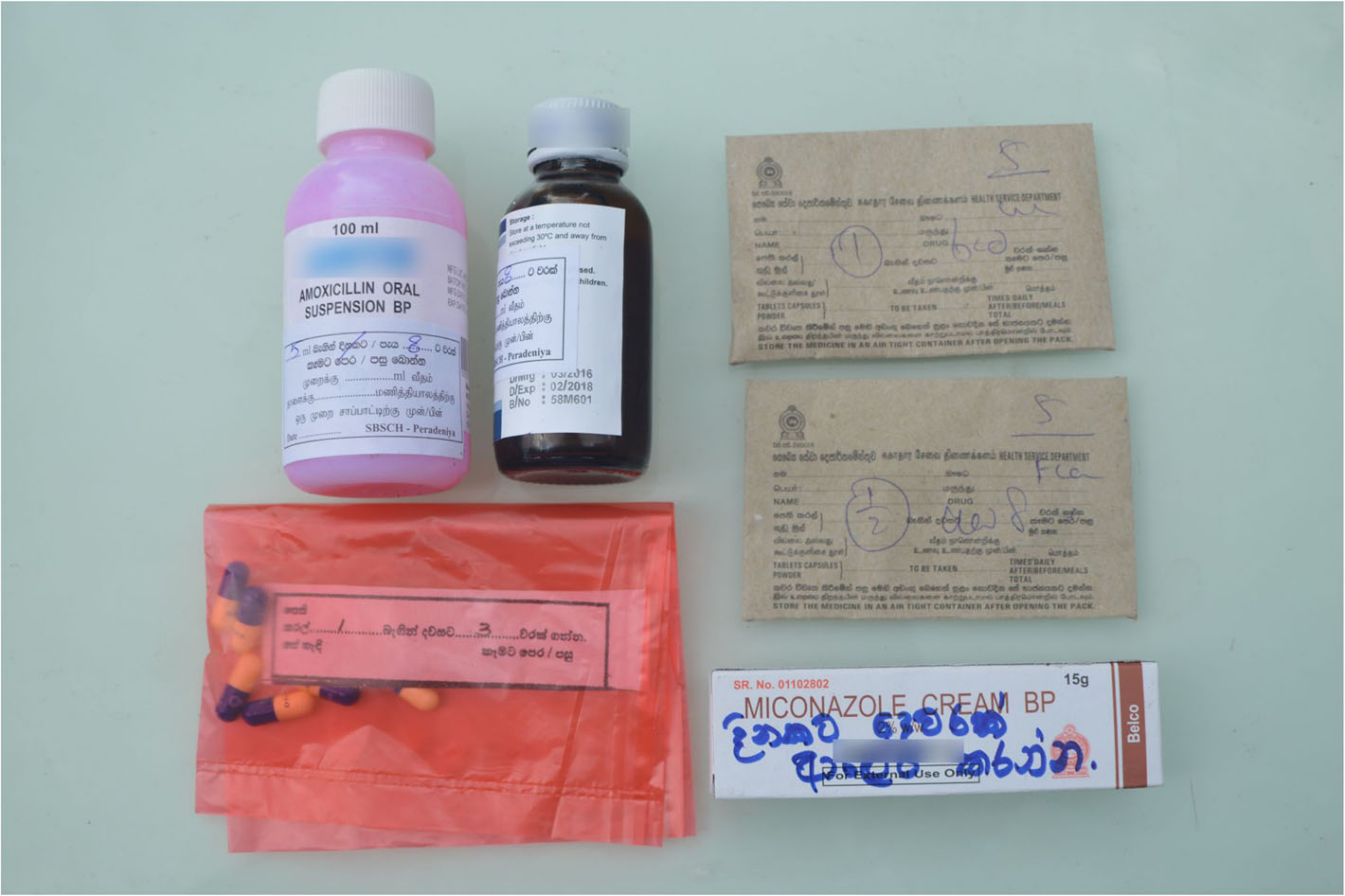
Example of medicines sourced from public facilities

**Fig. 3:**
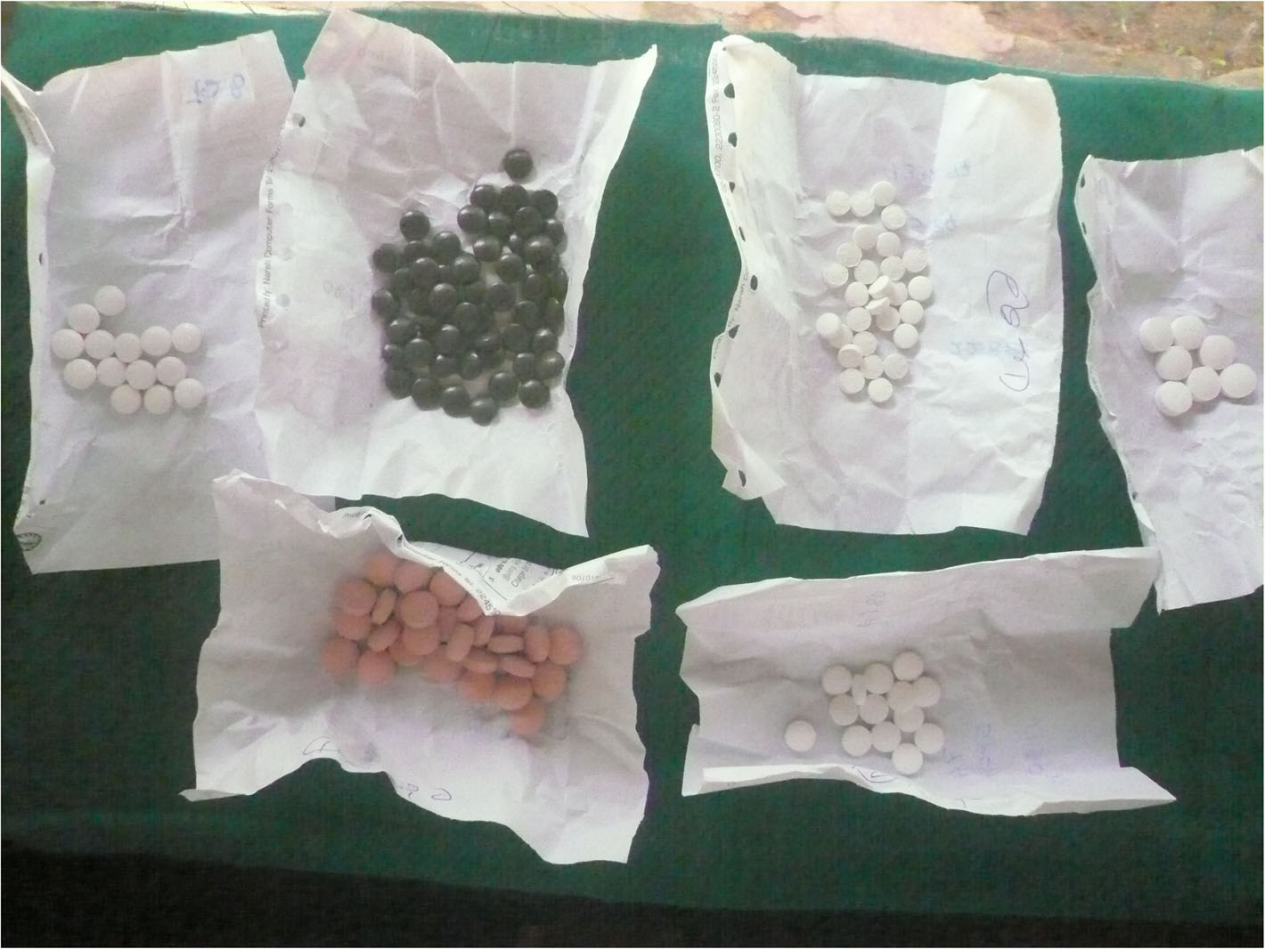
Example of unsatisfactory packaging using paper to wrap medicines

A greater proportion of western medicines sourced from private facilities were adequately packaged compared to those obtained from public facilities (87.7% vs 73.5%; OR 2.58, 95% CI 2.23, 2.99; Table 2). Medicines issued from both private and public sectors were mostly contained in envelopes, plastic bags or glass bottles (see Figs. 1 and 2). In some of the public facilities, the packaging was unsatisfactory. Although not very frequent, an example of unsatisfactory packaging included the use of a sheet of paper to wrap tablets and capsules (Fig. 3).

**Table 2:** Adequacy of packaging and labelling of western medicines issued by public and private facilities and by district

	Surveyed districts in Sri Lanka (*n*, %)						
	Ampara	Colombo	Kandy	Monaragala	Polonnaruwa	Rathnapura	Total
Medicines obtained from Public facilities							
Total medicines	453	284	336	439	478	425	2415
Adequate Label	106 (23.4)	118 (41.5)	68 (20.2)	100 (22.8)	152 (31.8)	117 (27.5)	661 (27.4)
Adequate Packaging	320 (70.6)	213 (75.0)	234 (69.6)	322 (73.3)	359 (75.4)	325 (76.5)	1773 (73.5)
Both Adequate	106 (23.4)	109 (38.4)	64 (19.0)	97 (22.1)	134 (28.0)	115 (27.1)	625 (25.9)
Medicines obtained from Private facilities							
Total medicines	232	687	549	473	287	590	2818
Adequate Label	128 (55.2)	440 (64.0)	288 (52.5)	229 (48.4)	138 (41.8)	247 (41.9)	1470 (52.5)
Adequate Packaging	217 (93.5)	655 (95.3)	492 (89.6)	412 (87.1)	251 (87.5)	444 (75.3)	2471 (87.7)
Both Adequate	127 (54.7)	438 (63.8)	266 (48.5)	219 (46.3)	127 (44.3)	234 (39.7)	1411 (50.1)

Public = public hospital, health centre or dispensary; Private = private health care provider or pharmacy

Private health facilities were more likely to have supplied medicines with adequate labels than public facilities (52.2% vs 27.4%; OR 2.90, 95% CI 2.57, 3.26); often with the label written on the envelope containing the medicine. The use of slips of paper inside medicine containers was common in public hospitals. In some public facilities where papers were used to wrap medicines, the label was usually written directly on the folded paper.

Only 25.9% of medicines issued by public sector facilities were determined to be both adequately packaged and adequately labelled compared to 50% of medicines obtained from the private sector. This observation of better packaging and labelling in the private sector was consistent across the six regions (Table 2). A greater proportion of medicines issued by public sector facilities were correctly labelled in Colombo region (41.5%) compared to other regions (20.2–31.8%).

## Discussion

The results of this study confirm previous observations of problems in dispensing practices in Sri Lanka and other LMICs, with notable differences in the quality of dispensing practices in the public and private sectors. In absolute terms the level of adequately labelled medicines was better than reported in studies conducted in Egypt, Northern Nigeria, Pakistan and Ethiopia [5–8], however labelling of medicines remains sub-optimal (<50%).

In general, medicines packaging was better than medicines labelling, however the criteria set for ‘adequate’ in both cases was quite low. An adequate label was one that was considered legible with the medicine name, dosage regimen and expiry date. This level of detail falls well short of current recommendations for labelling of outpatient medicines in high-income countries [12].

An adequate package was an envelope or closable container and containing only one medicine. We could not assess how long the medicines may have remained packaged this way; in busy health facilities staff may pre-package medicines into smaller units for individual patient use to facilitate the dispensing process. The implications of longer-term storage in envelopes and plastic bags on medicine potency are unclear. The use of folded sheets of paper to package some medicines from public facilities may indicate an acute shortage of proper material for dispensing at least in some facilities. Such practices may not be limited to public facilities, with a previous WHO situational analysis in Sri Lanka reporting instances of private pharmacies dispensing medicines in envelopes, sometimes made of old newspaper; and in a few of the facilities there was no labelling of medicines [13].

The high proportion of respondents indicating they understood the label and how to take the medicines was in contrast to the findings on adequate packaging and labelling. However, this is a consistent finding of published studies—in spite of poor labelling and presumably little time for contact with dispensing personnel. It is difficult to compare our results with other studies due to differences in definitions. In some studies, patients stating they knew the dose was considered a positive response [7], while in others, patients had to be able to repeat at least the dose and frequency of medication administration [6]. We did not have the level of detail to compare awareness of dosing for prescription medicines versus other medicine types in the household or levels of knowledge of dosage regimens for new medicines compared to medicines taken previously. Babu et al. reported that only 57.3% of patients taking antihypertensive medicines had adequate knowledge of their dosage schedule [14]. Adherence to prescribed medicine regimens is important if patients are to achieve the desired clinical benefits of their treatment. We made no assessment of the appropriateness of the medicines present in the household.

There were a number of limitations inherent in the design of this primarily descriptive survey. The assessment of medicines packaging and labelling occurred in family homes, and it is possible that medicines may have been re-packaged for convenience by household members and not kept in original packaging. Assessment of the household members’ understanding of how to take their medicines was limited to a simple yes/no response. There was no objective assessment of understanding of dosage instructions. Assessment of adequate labelling and packaging was made according to the WHO Manual for Household Surveys [11]. The standard for adequate labelling and packaging may not be considered adequate in other developed countries.

There has been increasing emphasis on effective drug regulatory procedures and good manufacturing practices (GMP) in medicines production and improved procedures for medicines storage and distribution to try to ensure that only quality-assured medicines are in circulation. However problems in dispensing practices remain [3, 15]. A notable feature of many of the studies is the short dispensing times and minimal interactions between dispensers and patients reported [13]. Syhakhang and colleagues found no differences in medicines adequately labelled in public and private pharmacy practices in one province of Lao PDR [16]. We cannot account for the differences observed in this study but there were suggestions of more acute shortages of appropriate dispensing materials in some of the public sector institutions.

There is evidence that training can improve dispensing practices [17]. However, education alone is unlikely to produce sustainable changes. Incentives, greater compliance with agreed professional pharmacy practice standards and technology solutions including electronic prescription systems that generate labels with standard patient information will all contribute to improved dispensing practices. Good Pharmacy Practice guidelines provide a quality management framework and a strategic plan for developing pharmacy services [18]. Preparing, obtaining, storing, securing, distributing, administering, dispensing and disposal of medical products are key functions. Much work and investment in staff and resources will be required in LMICs for pharmacists to fulfil these functions. However, as LMICS face ageing populations, the double burden of communicable and non-communicable chronic diseases, polypharmacy and more complex medicine regimens, the imperatives to provide quality pharmacy services grows.

## Conclusions

There were significant differences in the quality of labelling and packaging of western medicines in the public and private sectors in this study. In general, medicines packaging was better than medicines labelling, however the criteria set for ‘adequate’ in both cases were quite low, and well below those in current recommendations for Good Pharmacy Practices. Organizational constraints are likely to have contributed to the problems observed with inadequate dispensing resources (containers and labels), particularly in the public sector. Low staffing levels will also limit the ability of trained staff to provide instructions to patients and carers on how to take the medicines.

The results of this study highlight the need for training and continuing education in Good Pharmacy Practices in Sri Lanka and other LMICs with similar problems with packaging and labelling of dispensed medicines. The goal is to ensure that medicines are delivered to the patient with a label providing clear instructions on administration and a package that maintains the potency of the medicine up to the time of use. Ageing populations with more chronic diseases requiring polypharmacy and complex medicine regimens increase the need for appropriately packaged and labelled medicines.

## Additional file

Additional file 1: Excel data file with raw data used in this analysis. (XLSX 1051 kb)

## Abbreviations

CI: Confidence intervals
GMP: Good manufacturing practices
LMIC: Low and middle-income countries
OR: Odds ratio
WHO: World Health Organization
